# Supplementary material for: Pyruvate Dehydrogenase Kinase 1 inhibition mediated oxidative phosphorylation enhancement in cartilage promotes osteoarthritis progression
Source: BMC Musculoskelet Disord. 2023 Jul 20;24:597. doi: 10.1186/s12891-023-06585-6 (PMC10357736; doi:10.1186/s12891-023-06585-6)
Supplement: Supplementary file 1 — Additional file 1. [file 12891_2023_6585_MOESM1_ESM.zip › BP.Gsea.1653623667859/GOBP_CARBOHYDRATE_DERIVATIVE_BIOSYNTHETIC_PROCESS.html]

Details for gene set GOBP\_CARBOHYDRATE\_DERIVATIVE\_BIOSYNTHETIC\_PROCESS[GSEA]

|  || Dataset | 55457\_collapsed\_to\_symbols.cls.cls #OA\_versus\_normal.cls.cls #OA\_versus\_normal\_repos |
| Phenotype | cls.cls#OA\_versus\_normal\_repos |
| Upregulated in class | OA |
| GeneSet | GOBP\_CARBOHYDRATE\_DERIVATIVE\_BIOSYNTHETIC\_PROCESS |
| Enrichment Score (ES) | 0.26555216 |
| Normalized Enrichment Score (NES) | 1.5099187 |
| Nominal p-value | 0.0 |
| FDR q-value | 0.36010835 |
| FWER p-Value | 1.0 |
Table: GSEA Results Summary

  

Fig 1: Enrichment plot: GOBP\_CARBOHYDRATE\_DERIVATIVE\_BIOSYNTHETIC\_PROCESS      
 Profile of the Running ES Score & Positions of GeneSet Members on the Rank Ordered List

  

| SYMBOL | TITLE | RANK IN GENE LIST | RANK METRIC SCORE | RUNNING ES | CORE ENRICHMENT || 1 | C1GALT1C1 | C1GALT1 specific chaperone 1 [Source:HGNC Symbol;Acc:HGNC:24338] | 22 | 1.098 | 0.0057 | Yes |
| 2 | GPAA1 | glycosylphosphatidylinositol anchor attachment 1 [Source:HGNC Symbol;Acc:HGNC:4446] | 44 | 1.006 | 0.0109 | Yes |
| 3 | UXS1 | UDP-glucuronate decarboxylase 1 [Source:HGNC Symbol;Acc:HGNC:17729] | 91 | 0.926 | 0.0135 | Yes |
| 4 | DGUOK | deoxyguanosine kinase [Source:HGNC Symbol;Acc:HGNC:2858] | 96 | 0.917 | 0.0194 | Yes |
| 5 | B3GALNT1 | "beta-1,3-N-acetylgalactosaminyltransferase 1 (globoside blood group) [Source:HGNC Symbol;Acc:HGNC:918]" | 101 | 0.914 | 0.0253 | Yes |
| 6 | TMEM258 | transmembrane protein 258 [Source:HGNC Symbol;Acc:HGNC:1164] | 123 | 0.884 | 0.0296 | Yes |
| 7 | ADK | adenosine kinase [Source:HGNC Symbol;Acc:HGNC:257] | 127 | 0.878 | 0.0354 | Yes |
| 8 | ST8SIA1 | "ST8 alpha-N-acetyl-neuraminide alpha-2,8-sialyltransferase 1 [Source:HGNC Symbol;Acc:HGNC:10869]" | 133 | 0.873 | 0.0409 | Yes |
| 9 | MPC2 | mitochondrial pyruvate carrier 2 [Source:HGNC Symbol;Acc:HGNC:24515] | 140 | 0.870 | 0.0464 | Yes |
| 10 | PDHB | pyruvate dehydrogenase E1 subunit beta [Source:HGNC Symbol;Acc:HGNC:8808] | 149 | 0.862 | 0.0516 | Yes |
| 11 | GALNT11 | polypeptide N-acetylgalactosaminyltransferase 11 [Source:HGNC Symbol;Acc:HGNC:19875] | 164 | 0.845 | 0.0562 | Yes |
| 12 | EXT2 | exostosin glycosyltransferase 2 [Source:HGNC Symbol;Acc:HGNC:3513] | 170 | 0.838 | 0.0615 | Yes |
| 13 | NME7 | NME/NM23 family member 7 [Source:HGNC Symbol;Acc:HGNC:20461] | 178 | 0.835 | 0.0666 | Yes |
| 14 | PIGC | phosphatidylinositol glycan anchor biosynthesis class C [Source:HGNC Symbol;Acc:HGNC:8960] | 202 | 0.816 | 0.0704 | Yes |
| 15 | PANK2 | pantothenate kinase 2 [Source:HGNC Symbol;Acc:HGNC:15894] | 220 | 0.805 | 0.0745 | Yes |
| 16 | MGAT4C | MGAT4 family member C [Source:HGNC Symbol;Acc:HGNC:30871] | 234 | 0.796 | 0.0788 | Yes |
| 17 | MPI | mannose phosphate isomerase [Source:HGNC Symbol;Acc:HGNC:7216] | 255 | 0.781 | 0.0825 | Yes |
| 18 | B4GAT1 | "beta-1,4-glucuronyltransferase 1 [Source:HGNC Symbol;Acc:HGNC:15685]" | 282 | 0.764 | 0.0857 | Yes |
| 19 | TREM2 | triggering receptor expressed on myeloid cells 2 [Source:HGNC Symbol;Acc:HGNC:17761] | 342 | 0.726 | 0.0859 | Yes |
| 20 | EXTL2 | exostosin like glycosyltransferase 2 [Source:HGNC Symbol;Acc:HGNC:3516] | 357 | 0.722 | 0.0897 | Yes |
| 21 | NME5 | NME/NM23 family member 5 [Source:HGNC Symbol;Acc:HGNC:7853] | 362 | 0.720 | 0.0942 | Yes |
| 22 | PIGP | phosphatidylinositol glycan anchor biosynthesis class P [Source:HGNC Symbol;Acc:HGNC:3046] | 367 | 0.718 | 0.0988 | Yes |
| 23 | TREX1 | three prime repair exonuclease 1 [Source:HGNC Symbol;Acc:HGNC:12269] | 393 | 0.709 | 0.1016 | Yes |
| 24 | ATIC | 5-aminoimidazole-4-carboxamide ribonucleotide formyltransferase/IMP cyclohydrolase [Source:HGNC Symbol;Acc:HGNC:794] | 401 | 0.706 | 0.1059 | Yes |
| 25 | PSEN1 | presenilin 1 [Source:HGNC Symbol;Acc:HGNC:9508] | 425 | 0.695 | 0.1088 | Yes |
| 26 | DDOST | dolichyl-diphosphooligosaccharide--protein glycosyltransferase non-catalytic subunit [Source:HGNC Symbol;Acc:HGNC:2728] | 428 | 0.695 | 0.1133 | Yes |
| 27 | PIGT | phosphatidylinositol glycan anchor biosynthesis class T [Source:HGNC Symbol;Acc:HGNC:14938] | 444 | 0.686 | 0.1168 | Yes |
| 28 | PIGO | phosphatidylinositol glycan anchor biosynthesis class O [Source:HGNC Symbol;Acc:HGNC:23215] | 462 | 0.675 | 0.1200 | Yes |
| 29 | ATP5MC1 | ATP synthase membrane subunit c locus 1 [Source:HGNC Symbol;Acc:HGNC:841] | 465 | 0.674 | 0.1245 | Yes |
| 30 | AK1 | adenylate kinase 1 [Source:HGNC Symbol;Acc:HGNC:361] | 481 | 0.667 | 0.1278 | Yes |
| 31 | TYW1 | tRNA-yW synthesizing protein 1 homolog [Source:HGNC Symbol;Acc:HGNC:25598] | 523 | 0.650 | 0.1289 | Yes |
| 32 | ATP5PD | ATP synthase peripheral stalk subunit d [Source:HGNC Symbol;Acc:HGNC:845] | 564 | 0.635 | 0.1300 | Yes |
| 33 | DLD | dihydrolipoamide dehydrogenase [Source:HGNC Symbol;Acc:HGNC:2898] | 565 | 0.635 | 0.1343 | Yes |
| 34 | PAPSS2 | 3'-phosphoadenosine 5'-phosphosulfate synthase 2 [Source:HGNC Symbol;Acc:HGNC:8604] | 592 | 0.627 | 0.1365 | Yes |
| 35 | ANTKMT | adenine nucleotide translocase lysine methyltransferase [Source:HGNC Symbol;Acc:HGNC:14152] | 618 | 0.618 | 0.1387 | Yes |
| 36 | MGAT4A | "alpha-1,3-mannosyl-glycoprotein 4-beta-N-acetylglucosaminyltransferase A [Source:HGNC Symbol;Acc:HGNC:7047]" | 657 | 0.604 | 0.1398 | Yes |
| 37 | NCSTN | nicastrin [Source:HGNC Symbol;Acc:HGNC:17091] | 666 | 0.601 | 0.1432 | Yes |
| 38 | NME4 | NME/NM23 nucleoside diphosphate kinase 4 [Source:HGNC Symbol;Acc:HGNC:7852] | 677 | 0.599 | 0.1465 | Yes |
| 39 | DOLK | dolichol kinase [Source:HGNC Symbol;Acc:HGNC:23406] | 683 | 0.598 | 0.1502 | Yes |
| 40 | DMAC2L | distal membrane arm assembly component 2 like [Source:HGNC Symbol;Acc:HGNC:18799] | 695 | 0.595 | 0.1533 | Yes |
| 41 | ALG6 | "ALG6 alpha-1,3-glucosyltransferase [Source:HGNC Symbol;Acc:HGNC:23157]" | 696 | 0.595 | 0.1574 | Yes |
| 42 | PAPSS1 | 3'-phosphoadenosine 5'-phosphosulfate synthase 1 [Source:HGNC Symbol;Acc:HGNC:8603] | 703 | 0.593 | 0.1609 | Yes |
| 43 | PPCS | phosphopantothenoylcysteine synthetase [Source:HGNC Symbol;Acc:HGNC:25686] | 716 | 0.589 | 0.1640 | Yes |
| 44 | DAD1 | defender against cell death 1 [Source:HGNC Symbol;Acc:HGNC:2664] | 737 | 0.585 | 0.1664 | Yes |
| 45 | CANT1 | calcium activated nucleotidase 1 [Source:HGNC Symbol;Acc:HGNC:19721] | 783 | 0.573 | 0.1666 | Yes |
| 46 | HSD17B12 | hydroxysteroid 17-beta dehydrogenase 12 [Source:HGNC Symbol;Acc:HGNC:18646] | 795 | 0.570 | 0.1696 | Yes |
| 47 | PIGF | phosphatidylinositol glycan anchor biosynthesis class F [Source:HGNC Symbol;Acc:HGNC:8962] | 798 | 0.570 | 0.1734 | Yes |
| 48 | AKR1A1 | aldo-keto reductase family 1 member A1 [Source:HGNC Symbol;Acc:HGNC:380] | 808 | 0.567 | 0.1765 | Yes |
| 49 | ATP5PO | ATP synthase peripheral stalk subunit OSCP [Source:HGNC Symbol;Acc:HGNC:850] | 840 | 0.558 | 0.1778 | Yes |
| 50 | SLC2A10 | solute carrier family 2 member 10 [Source:HGNC Symbol;Acc:HGNC:13444] | 874 | 0.550 | 0.1789 | Yes |
| 51 | NME3 | NME/NM23 nucleoside diphosphate kinase 3 [Source:HGNC Symbol;Acc:HGNC:7851] | 876 | 0.549 | 0.1826 | Yes |
| 52 | ALG8 | "ALG8 alpha-1,3-glucosyltransferase [Source:HGNC Symbol;Acc:HGNC:23161]" | 900 | 0.543 | 0.1844 | Yes |
| 53 | DCK | deoxycytidine kinase [Source:HGNC Symbol;Acc:HGNC:2704] | 920 | 0.537 | 0.1865 | Yes |
| 54 | ALG5 | ALG5 dolichyl-phosphate beta-glucosyltransferase [Source:HGNC Symbol;Acc:HGNC:20266] | 945 | 0.529 | 0.1882 | Yes |
| 55 | LCMT2 | leucine carboxyl methyltransferase 2 [Source:HGNC Symbol;Acc:HGNC:17558] | 946 | 0.529 | 0.1918 | Yes |
| 56 | B3GALT4 | "beta-1,3-galactosyltransferase 4 [Source:HGNC Symbol;Acc:HGNC:919]" | 962 | 0.526 | 0.1942 | Yes |
| 57 | ACOT7 | acyl-CoA thioesterase 7 [Source:HGNC Symbol;Acc:HGNC:24157] | 971 | 0.524 | 0.1971 | Yes |
| 58 | CHST12 | carbohydrate sulfotransferase 12 [Source:HGNC Symbol;Acc:HGNC:17423] | 985 | 0.521 | 0.1996 | Yes |
| 59 | HYAL1 | hyaluronidase 1 [Source:HGNC Symbol;Acc:HGNC:5320] | 997 | 0.518 | 0.2023 | Yes |
| 60 | ACAT1 | acetyl-CoA acetyltransferase 1 [Source:HGNC Symbol;Acc:HGNC:93] | 1017 | 0.513 | 0.2042 | Yes |
| 61 | PPT1 | palmitoyl-protein thioesterase 1 [Source:HGNC Symbol;Acc:HGNC:9325] | 1042 | 0.508 | 0.2058 | Yes |
| 62 | TECR | "trans-2,3-enoyl-CoA reductase [Source:HGNC Symbol;Acc:HGNC:4551]" | 1047 | 0.508 | 0.2089 | Yes |
| 63 | PIGV | phosphatidylinositol glycan anchor biosynthesis class V [Source:HGNC Symbol;Acc:HGNC:26031] | 1064 | 0.503 | 0.2110 | Yes |
| 64 | MAN2A1 | mannosidase alpha class 2A member 1 [Source:HGNC Symbol;Acc:HGNC:6824] | 1069 | 0.501 | 0.2141 | Yes |
| 65 | ACOT8 | acyl-CoA thioesterase 8 [Source:HGNC Symbol;Acc:HGNC:15919] | 1088 | 0.500 | 0.2161 | Yes |
| 66 | DCTD | dCMP deaminase [Source:HGNC Symbol;Acc:HGNC:2710] | 1107 | 0.494 | 0.2180 | Yes |
| 67 | C1GALT1 | "core 1 synthase, glycoprotein-N-acetylgalactosamine 3-beta-galactosyltransferase 1 [Source:HGNC Symbol;Acc:HGNC:24337]" | 1110 | 0.494 | 0.2212 | Yes |
| 68 | GALNT10 | polypeptide N-acetylgalactosaminyltransferase 10 [Source:HGNC Symbol;Acc:HGNC:19873] | 1173 | 0.481 | 0.2195 | Yes |
| 69 | APRT | adenine phosphoribosyltransferase [Source:HGNC Symbol;Acc:HGNC:626] | 1178 | 0.481 | 0.2225 | Yes |
| 70 | MAN1C1 | mannosidase alpha class 1C member 1 [Source:HGNC Symbol;Acc:HGNC:19080] | 1275 | 0.463 | 0.2179 | Yes |
| 71 | ATP5PB | ATP synthase peripheral stalk-membrane subunit b [Source:HGNC Symbol;Acc:HGNC:840] | 1293 | 0.459 | 0.2196 | Yes |
| 72 | RPN2 | ribophorin II [Source:HGNC Symbol;Acc:HGNC:10382] | 1294 | 0.459 | 0.2228 | Yes |
| 73 | ATP5MF | ATP synthase membrane subunit f [Source:HGNC Symbol;Acc:HGNC:848] | 1330 | 0.453 | 0.2230 | Yes |
| 74 | ADCY7 | adenylate cyclase 7 [Source:HGNC Symbol;Acc:HGNC:238] | 1408 | 0.442 | 0.2199 | Yes |
| 75 | B4GALT7 | "beta-1,4-galactosyltransferase 7 [Source:HGNC Symbol;Acc:HGNC:930]" | 1411 | 0.442 | 0.2227 | Yes |
| 76 | B3GNT2 | "UDP-GlcNAc:betaGal beta-1,3-N-acetylglucosaminyltransferase 2 [Source:HGNC Symbol;Acc:HGNC:15629]" | 1427 | 0.439 | 0.2245 | Yes |
| 77 | NME2 | NME/NM23 nucleoside diphosphate kinase 2 [Source:HGNC Symbol;Acc:HGNC:7850] | 1428 | 0.439 | 0.2275 | Yes |
| 78 | UBE2J1 | ubiquitin conjugating enzyme E2 J1 [Source:HGNC Symbol;Acc:HGNC:17598] | 1433 | 0.438 | 0.2301 | Yes |
| 79 | ANGPT1 | angiopoietin 1 [Source:HGNC Symbol;Acc:HGNC:484] | 1467 | 0.433 | 0.2304 | Yes |
| 80 | ALG9 | "ALG9 alpha-1,2-mannosyltransferase [Source:HGNC Symbol;Acc:HGNC:15672]" | 1487 | 0.431 | 0.2318 | Yes |
| 81 | ABO | "ABO, alpha 1-3-N-acetylgalactosaminyltransferase and alpha 1-3-galactosyltransferase [Source:HGNC Symbol;Acc:HGNC:79]" | 1495 | 0.429 | 0.2342 | Yes |
| 82 | B4GALT6 | "beta-1,4-galactosyltransferase 6 [Source:HGNC Symbol;Acc:HGNC:929]" | 1500 | 0.429 | 0.2368 | Yes |
| 83 | FUT8 | fucosyltransferase 8 [Source:HGNC Symbol;Acc:HGNC:4019] | 1565 | 0.418 | 0.2345 | Yes |
| 84 | TM9SF2 | transmembrane 9 superfamily member 2 [Source:HGNC Symbol;Acc:HGNC:11865] | 1582 | 0.416 | 0.2361 | Yes |
| 85 | NDST2 | N-deacetylase and N-sulfotransferase 2 [Source:HGNC Symbol;Acc:HGNC:7681] | 1587 | 0.415 | 0.2386 | Yes |
| 86 | ADCY1 | adenylate cyclase 1 [Source:HGNC Symbol;Acc:HGNC:232] | 1589 | 0.415 | 0.2413 | Yes |
| 87 | AMDHD2 | amidohydrolase domain containing 2 [Source:HGNC Symbol;Acc:HGNC:24262] | 1595 | 0.415 | 0.2437 | Yes |
| 88 | NAGPA | N-acetylglucosamine-1-phosphodiester alpha-N-acetylglucosaminidase [Source:HGNC Symbol;Acc:HGNC:17378] | 1608 | 0.412 | 0.2456 | Yes |
| 89 | GUK1 | guanylate kinase 1 [Source:HGNC Symbol;Acc:HGNC:4693] | 1640 | 0.407 | 0.2458 | Yes |
| 90 | TMEM59 | transmembrane protein 59 [Source:HGNC Symbol;Acc:HGNC:1239] | 1644 | 0.407 | 0.2484 | Yes |
| 91 | PGAP2 | post-GPI attachment to proteins 2 [Source:HGNC Symbol;Acc:HGNC:17893] | 1669 | 0.403 | 0.2492 | Yes |
| 92 | MPPE1 | metallophosphoesterase 1 [Source:HGNC Symbol;Acc:HGNC:15988] | 1675 | 0.402 | 0.2515 | Yes |
| 93 | GCNT3 | "glucosaminyl (N-acetyl) transferase 3, mucin type [Source:HGNC Symbol;Acc:HGNC:4205]" | 1683 | 0.401 | 0.2537 | Yes |
| 94 | COG7 | component of oligomeric golgi complex 7 [Source:HGNC Symbol;Acc:HGNC:18622] | 1691 | 0.400 | 0.2559 | Yes |
| 95 | PDK2 | pyruvate dehydrogenase kinase 2 [Source:HGNC Symbol;Acc:HGNC:8810] | 1772 | 0.388 | 0.2521 | Yes |
| 96 | AMPD1 | adenosine monophosphate deaminase 1 [Source:HGNC Symbol;Acc:HGNC:468] | 1789 | 0.385 | 0.2534 | Yes |
| 97 | SNCA | synuclein alpha [Source:HGNC Symbol;Acc:HGNC:11138] | 1800 | 0.384 | 0.2552 | Yes |
| 98 | STOML2 | stomatin like 2 [Source:HGNC Symbol;Acc:HGNC:14559] | 1819 | 0.382 | 0.2564 | Yes |
| 99 | ST8SIA4 | "ST8 alpha-N-acetyl-neuraminide alpha-2,8-sialyltransferase 4 [Source:HGNC Symbol;Acc:HGNC:10871]" | 1850 | 0.379 | 0.2565 | Yes |
| 100 | XYLT2 | xylosyltransferase 2 [Source:HGNC Symbol;Acc:HGNC:15517] | 1938 | 0.368 | 0.2521 | Yes |
| 101 | ADA2 | adenosine deaminase 2 [Source:HGNC Symbol;Acc:HGNC:1839] | 1955 | 0.367 | 0.2533 | Yes |
| 102 | ELOVL4 | ELOVL fatty acid elongase 4 [Source:HGNC Symbol;Acc:HGNC:14415] | 2017 | 0.360 | 0.2508 | Yes |
| 103 | GCNT1 | glucosaminyl (N-acetyl) transferase 1 [Source:HGNC Symbol;Acc:HGNC:4203] | 2035 | 0.357 | 0.2519 | Yes |
| 104 | CCL19 | C-C motif chemokine ligand 19 [Source:HGNC Symbol;Acc:HGNC:10617] | 2060 | 0.355 | 0.2524 | Yes |
| 105 | PINK1 | PTEN induced kinase 1 [Source:HGNC Symbol;Acc:HGNC:14581] | 2097 | 0.350 | 0.2519 | Yes |
| 106 | PRPSAP1 | phosphoribosyl pyrophosphate synthetase associated protein 1 [Source:HGNC Symbol;Acc:HGNC:9466] | 2112 | 0.348 | 0.2531 | Yes |
| 107 | PDK1 | pyruvate dehydrogenase kinase 1 [Source:HGNC Symbol;Acc:HGNC:8809] | 2124 | 0.347 | 0.2546 | Yes |
| 108 | ATP5F1C | ATP synthase F1 subunit gamma [Source:HGNC Symbol;Acc:HGNC:833] | 2190 | 0.339 | 0.2517 | Yes |
| 109 | PPCDC | phosphopantothenoylcysteine decarboxylase [Source:HGNC Symbol;Acc:HGNC:28107] | 2251 | 0.332 | 0.2491 | Yes |
| 110 | CHPF2 | chondroitin polymerizing factor 2 [Source:HGNC Symbol;Acc:HGNC:29270] | 2281 | 0.329 | 0.2490 | Yes |
| 111 | IGF1 | insulin like growth factor 1 [Source:HGNC Symbol;Acc:HGNC:5464] | 2286 | 0.328 | 0.2509 | Yes |
| 112 | FUT4 | fucosyltransferase 4 [Source:HGNC Symbol;Acc:HGNC:4015] | 2318 | 0.324 | 0.2506 | Yes |
| 113 | PGAP3 | post-GPI attachment to proteins phospholipase 3 [Source:HGNC Symbol;Acc:HGNC:23719] | 2322 | 0.324 | 0.2526 | Yes |
| 114 | FUT7 | fucosyltransferase 7 [Source:HGNC Symbol;Acc:HGNC:4018] | 2343 | 0.322 | 0.2532 | Yes |
| 115 | ATP5MC2 | ATP synthase membrane subunit c locus 2 [Source:HGNC Symbol;Acc:HGNC:842] | 2371 | 0.318 | 0.2532 | Yes |
| 116 | EGF | epidermal growth factor [Source:HGNC Symbol;Acc:HGNC:3229] | 2399 | 0.315 | 0.2531 | Yes |
| 117 | PDHA1 | pyruvate dehydrogenase E1 subunit alpha 1 [Source:HGNC Symbol;Acc:HGNC:8806] | 2433 | 0.310 | 0.2526 | Yes |
| 118 | MLEC | malectin [Source:HGNC Symbol;Acc:HGNC:28973] | 2436 | 0.310 | 0.2546 | Yes |
| 119 | SPHK2 | sphingosine kinase 2 [Source:HGNC Symbol;Acc:HGNC:18859] | 2478 | 0.305 | 0.2533 | Yes |
| 120 | PIGN | phosphatidylinositol glycan anchor biosynthesis class N [Source:HGNC Symbol;Acc:HGNC:8967] | 2484 | 0.304 | 0.2550 | Yes |
| 121 | TGFB1 | transforming growth factor beta 1 [Source:HGNC Symbol;Acc:HGNC:11766] | 2490 | 0.303 | 0.2567 | Yes |
| 122 | ITM2C | integral membrane protein 2C [Source:HGNC Symbol;Acc:HGNC:6175] | 2495 | 0.303 | 0.2584 | Yes |
| 123 | CSGALNACT1 | chondroitin sulfate N-acetylgalactosaminyltransferase 1 [Source:HGNC Symbol;Acc:HGNC:24290] | 2507 | 0.302 | 0.2596 | Yes |
| 124 | ACSBG1 | acyl-CoA synthetase bubblegum family member 1 [Source:HGNC Symbol;Acc:HGNC:29567] | 2520 | 0.301 | 0.2607 | Yes |
| 125 | SLC35C2 | solute carrier family 35 member C2 [Source:HGNC Symbol;Acc:HGNC:17117] | 2563 | 0.295 | 0.2593 | Yes |
| 126 | PIGH | phosphatidylinositol glycan anchor biosynthesis class H [Source:HGNC Symbol;Acc:HGNC:8964] | 2573 | 0.294 | 0.2606 | Yes |
| 127 | IL15 | interleukin 15 [Source:HGNC Symbol;Acc:HGNC:5977] | 2599 | 0.291 | 0.2605 | Yes |
| 128 | DPM3 | "dolichyl-phosphate mannosyltransferase subunit 3, regulatory [Source:HGNC Symbol;Acc:HGNC:3007]" | 2618 | 0.289 | 0.2611 | Yes |
| 129 | HS3ST3A1 | heparan sulfate-glucosamine 3-sulfotransferase 3A1 [Source:HGNC Symbol;Acc:HGNC:5196] | 2637 | 0.286 | 0.2616 | Yes |
| 130 | ST6GALNAC5 | "ST6 N-acetylgalactosaminide alpha-2,6-sialyltransferase 5 [Source:HGNC Symbol;Acc:HGNC:19342]" | 2641 | 0.286 | 0.2633 | Yes |
| 131 | ATP5MC3 | ATP synthase membrane subunit c locus 3 [Source:HGNC Symbol;Acc:HGNC:843] | 2643 | 0.286 | 0.2651 | Yes |
| 132 | PIGB | phosphatidylinositol glycan anchor biosynthesis class B [Source:HGNC Symbol;Acc:HGNC:8959] | 2663 | 0.284 | 0.2656 | Yes |
| 133 | ATP5F1D | ATP synthase F1 subunit delta [Source:HGNC Symbol;Acc:HGNC:837] | 2701 | 0.279 | 0.2645 | No |
| 134 | ATP6V0C | ATPase H+ transporting V0 subunit c [Source:HGNC Symbol;Acc:HGNC:855] | 2790 | 0.271 | 0.2592 | No |
| 135 | PIGK | phosphatidylinositol glycan anchor biosynthesis class K [Source:HGNC Symbol;Acc:HGNC:8965] | 2801 | 0.270 | 0.2603 | No |
| 136 | GUCY2D | "guanylate cyclase 2D, retinal [Source:HGNC Symbol;Acc:HGNC:4689]" | 2832 | 0.267 | 0.2597 | No |
| 137 | PRPSAP2 | phosphoribosyl pyrophosphate synthetase associated protein 2 [Source:HGNC Symbol;Acc:HGNC:9467] | 2855 | 0.265 | 0.2597 | No |
| 138 | IMPDH2 | inosine monophosphate dehydrogenase 2 [Source:HGNC Symbol;Acc:HGNC:6053] | 2867 | 0.264 | 0.2606 | No |
| 139 | B3GALT2 | "beta-1,3-galactosyltransferase 2 [Source:HGNC Symbol;Acc:HGNC:917]" | 2883 | 0.262 | 0.2612 | No |
| 140 | UMPS | uridine monophosphate synthetase [Source:HGNC Symbol;Acc:HGNC:12563] | 2936 | 0.257 | 0.2588 | No |
| 141 | ATP5MG | ATP synthase membrane subunit g [Source:HGNC Symbol;Acc:HGNC:14247] | 2941 | 0.256 | 0.2602 | No |
| 142 | GALNT7 | polypeptide N-acetylgalactosaminyltransferase 7 [Source:HGNC Symbol;Acc:HGNC:4129] | 2954 | 0.255 | 0.2610 | No |
| 143 | GAL3ST4 | galactose-3-O-sulfotransferase 4 [Source:HGNC Symbol;Acc:HGNC:24145] | 2979 | 0.252 | 0.2608 | No |
| 144 | GUCY2C | guanylate cyclase 2C [Source:HGNC Symbol;Acc:HGNC:4688] | 2982 | 0.252 | 0.2623 | No |
| 145 | B3GAT3 | "beta-1,3-glucuronyltransferase 3 [Source:HGNC Symbol;Acc:HGNC:923]" | 2987 | 0.251 | 0.2637 | No |
| 146 | ATP5F1B | ATP synthase F1 subunit beta [Source:HGNC Symbol;Acc:HGNC:830] | 3024 | 0.247 | 0.2625 | No |
| 147 | HEXA | hexosaminidase subunit alpha [Source:HGNC Symbol;Acc:HGNC:4878] | 3025 | 0.247 | 0.2642 | No |
| 148 | GK2 | glycerol kinase 2 [Source:HGNC Symbol;Acc:HGNC:4291] | 3042 | 0.246 | 0.2646 | No |
| 149 | PLOD1 | "procollagen-lysine,2-oxoglutarate 5-dioxygenase 1 [Source:HGNC Symbol;Acc:HGNC:9081]" | 3155 | 0.235 | 0.2571 | No |
| 150 | UST | uronyl 2-sulfotransferase [Source:HGNC Symbol;Acc:HGNC:17223] | 3180 | 0.233 | 0.2568 | No |
| 151 | SCCPDH | saccharopine dehydrogenase (putative) [Source:HGNC Symbol;Acc:HGNC:24275] | 3197 | 0.231 | 0.2571 | No |
| 152 | POGLUT2 | protein O-glucosyltransferase 2 [Source:HGNC Symbol;Acc:HGNC:19350] | 3238 | 0.227 | 0.2554 | No |
| 153 | ELOVL6 | ELOVL fatty acid elongase 6 [Source:HGNC Symbol;Acc:HGNC:15829] | 3253 | 0.226 | 0.2558 | No |
| 154 | NPPC | natriuretic peptide C [Source:HGNC Symbol;Acc:HGNC:7941] | 3276 | 0.223 | 0.2556 | No |
| 155 | PID1 | phosphotyrosine interaction domain containing 1 [Source:HGNC Symbol;Acc:HGNC:26084] | 3280 | 0.222 | 0.2568 | No |
| 156 | EXT1 | exostosin glycosyltransferase 1 [Source:HGNC Symbol;Acc:HGNC:3512] | 3321 | 0.219 | 0.2551 | No |
| 157 | MTAP | methylthioadenosine phosphorylase [Source:HGNC Symbol;Acc:HGNC:7413] | 3324 | 0.219 | 0.2565 | No |
| 158 | ST3GAL6 | "ST3 beta-galactoside alpha-2,3-sialyltransferase 6 [Source:HGNC Symbol;Acc:HGNC:18080]" | 3339 | 0.218 | 0.2568 | No |
| 159 | HS2ST1 | heparan sulfate 2-O-sulfotransferase 1 [Source:HGNC Symbol;Acc:HGNC:5193] | 3364 | 0.215 | 0.2563 | No |
| 160 | B4GALT4 | "beta-1,4-galactosyltransferase 4 [Source:HGNC Symbol;Acc:HGNC:927]" | 3407 | 0.211 | 0.2544 | No |
| 161 | PARP1 | poly(ADP-ribose) polymerase 1 [Source:HGNC Symbol;Acc:HGNC:270] | 3410 | 0.210 | 0.2557 | No |
| 162 | MAN1B1 | mannosidase alpha class 1B member 1 [Source:HGNC Symbol;Acc:HGNC:6823] | 3447 | 0.207 | 0.2542 | No |
| 163 | TNIP1 | TNFAIP3 interacting protein 1 [Source:HGNC Symbol;Acc:HGNC:16903] | 3459 | 0.206 | 0.2547 | No |
| 164 | NDST4 | N-deacetylase and N-sulfotransferase 4 [Source:HGNC Symbol;Acc:HGNC:20779] | 3497 | 0.202 | 0.2531 | No |
| 165 | COASY | Coenzyme A synthase [Source:HGNC Symbol;Acc:HGNC:29932] | 3498 | 0.202 | 0.2545 | No |
| 166 | ST6GAL1 | "ST6 beta-galactoside alpha-2,6-sialyltransferase 1 [Source:HGNC Symbol;Acc:HGNC:10860]" | 3502 | 0.202 | 0.2556 | No |
| 167 | RRM2 | ribonucleotide reductase regulatory subunit M2 [Source:HGNC Symbol;Acc:HGNC:10452] | 3505 | 0.202 | 0.2568 | No |
| 168 | NT5E | 5'-nucleotidase ecto [Source:HGNC Symbol;Acc:HGNC:8021] | 3515 | 0.201 | 0.2575 | No |
| 169 | VCP | valosin containing protein [Source:HGNC Symbol;Acc:HGNC:12666] | 3518 | 0.201 | 0.2587 | No |
| 170 | DTYMK | deoxythymidylate kinase [Source:HGNC Symbol;Acc:HGNC:3061] | 3538 | 0.199 | 0.2585 | No |
| 171 | PLOD3 | "procollagen-lysine,2-oxoglutarate 5-dioxygenase 3 [Source:HGNC Symbol;Acc:HGNC:9083]" | 3614 | 0.192 | 0.2538 | No |
| 172 | POMGNT1 | "protein O-linked mannose N-acetylglucosaminyltransferase 1 (beta 1,2-) [Source:HGNC Symbol;Acc:HGNC:19139]" | 3641 | 0.191 | 0.2530 | No |
| 173 | ST3GAL5 | "ST3 beta-galactoside alpha-2,3-sialyltransferase 5 [Source:HGNC Symbol;Acc:HGNC:10872]" | 3667 | 0.188 | 0.2523 | No |
| 174 | PRPS1 | phosphoribosyl pyrophosphate synthetase 1 [Source:HGNC Symbol;Acc:HGNC:9462] | 3717 | 0.184 | 0.2496 | No |
| 175 | ALG12 | "ALG12 alpha-1,6-mannosyltransferase [Source:HGNC Symbol;Acc:HGNC:19358]" | 3723 | 0.183 | 0.2504 | No |
| 176 | PLCB1 | phospholipase C beta 1 [Source:HGNC Symbol;Acc:HGNC:15917] | 3724 | 0.183 | 0.2517 | No |
| 177 | CEMIP | cell migration inducing hyaluronidase 1 [Source:HGNC Symbol;Acc:HGNC:29213] | 3747 | 0.182 | 0.2511 | No |
| 178 | TBPL1 | TATA-box binding protein like 1 [Source:HGNC Symbol;Acc:HGNC:11589] | 3794 | 0.178 | 0.2487 | No |
| 179 | PYGL | glycogen phosphorylase L [Source:HGNC Symbol;Acc:HGNC:9725] | 3826 | 0.174 | 0.2473 | No |
| 180 | AK5 | adenylate kinase 5 [Source:HGNC Symbol;Acc:HGNC:365] | 3841 | 0.172 | 0.2474 | No |
| 181 | GCNT4 | glucosaminyl (N-acetyl) transferase 4 [Source:HGNC Symbol;Acc:HGNC:17973] | 3858 | 0.169 | 0.2473 | No |
| 182 | DPY19L1 | dpy-19 like C-mannosyltransferase 1 [Source:HGNC Symbol;Acc:HGNC:22205] | 3875 | 0.167 | 0.2471 | No |
| 183 | XYLT1 | xylosyltransferase 1 [Source:HGNC Symbol;Acc:HGNC:15516] | 3916 | 0.163 | 0.2450 | No |
| 184 | MAGT1 | magnesium transporter 1 [Source:HGNC Symbol;Acc:HGNC:28880] | 3920 | 0.163 | 0.2459 | No |
| 185 | TUSC3 | tumor suppressor candidate 3 [Source:HGNC Symbol;Acc:HGNC:30242] | 3940 | 0.161 | 0.2454 | No |
| 186 | GUCY1B1 | guanylate cyclase 1 soluble subunit beta 1 [Source:HGNC Symbol;Acc:HGNC:4687] | 3962 | 0.159 | 0.2448 | No |
| 187 | TRAK1 | trafficking kinesin protein 1 [Source:HGNC Symbol;Acc:HGNC:29947] | 3964 | 0.159 | 0.2458 | No |
| 188 | TPI1 | triosephosphate isomerase 1 [Source:HGNC Symbol;Acc:HGNC:12009] | 4059 | 0.150 | 0.2393 | No |
| 189 | ALG3 | "ALG3 alpha-1,3- mannosyltransferase [Source:HGNC Symbol;Acc:HGNC:23056]" | 4096 | 0.146 | 0.2374 | No |
| 190 | ATP5F1A | ATP synthase F1 subunit alpha [Source:HGNC Symbol;Acc:HGNC:823] | 4137 | 0.143 | 0.2352 | No |
| 191 | GMDS | "GDP-mannose 4,6-dehydratase [Source:HGNC Symbol;Acc:HGNC:4369]" | 4151 | 0.142 | 0.2351 | No |
| 192 | ST8SIA2 | "ST8 alpha-N-acetyl-neuraminide alpha-2,8-sialyltransferase 2 [Source:HGNC Symbol;Acc:HGNC:10870]" | 4153 | 0.142 | 0.2360 | No |
| 193 | AMPD3 | adenosine monophosphate deaminase 3 [Source:HGNC Symbol;Acc:HGNC:470] | 4174 | 0.140 | 0.2353 | No |
| 194 | AK2 | adenylate kinase 2 [Source:HGNC Symbol;Acc:HGNC:362] | 4179 | 0.140 | 0.2359 | No |
| 195 | RAMP1 | receptor activity modifying protein 1 [Source:HGNC Symbol;Acc:HGNC:9843] | 4201 | 0.138 | 0.2352 | No |
| 196 | ITM2A | integral membrane protein 2A [Source:HGNC Symbol;Acc:HGNC:6173] | 4203 | 0.138 | 0.2360 | No |
| 197 | PMM1 | phosphomannomutase 1 [Source:HGNC Symbol;Acc:HGNC:9114] | 4238 | 0.134 | 0.2342 | No |
| 198 | NME6 | NME/NM23 nucleoside diphosphate kinase 6 [Source:HGNC Symbol;Acc:HGNC:20567] | 4257 | 0.132 | 0.2337 | No |
| 199 | MAN1A2 | mannosidase alpha class 1A member 2 [Source:HGNC Symbol;Acc:HGNC:6822] | 4272 | 0.131 | 0.2334 | No |
| 200 | ALDOA | "aldolase, fructose-bisphosphate A [Source:HGNC Symbol;Acc:HGNC:414]" | 4289 | 0.129 | 0.2330 | No |
| 201 | TYMS | thymidylate synthetase [Source:HGNC Symbol;Acc:HGNC:12441] | 4328 | 0.126 | 0.2308 | No |
| 202 | CLTC | clathrin heavy chain [Source:HGNC Symbol;Acc:HGNC:2092] | 4330 | 0.126 | 0.2316 | No |
| 203 | DHDDS | dehydrodolichyl diphosphate synthase subunit [Source:HGNC Symbol;Acc:HGNC:20603] | 4356 | 0.124 | 0.2304 | No |
| 204 | PRKAG2 | protein kinase AMP-activated non-catalytic subunit gamma 2 [Source:HGNC Symbol;Acc:HGNC:9386] | 4362 | 0.123 | 0.2309 | No |
| 205 | PRPS2 | phosphoribosyl pyrophosphate synthetase 2 [Source:HGNC Symbol;Acc:HGNC:9465] | 4365 | 0.123 | 0.2316 | No |
| 206 | GOLPH3 | golgi phosphoprotein 3 [Source:HGNC Symbol;Acc:HGNC:15452] | 4420 | 0.119 | 0.2280 | No |
| 207 | GMPS | guanine monophosphate synthase [Source:HGNC Symbol;Acc:HGNC:4378] | 4498 | 0.113 | 0.2226 | No |
| 208 | RRM1 | ribonucleotide reductase catalytic subunit M1 [Source:HGNC Symbol;Acc:HGNC:10451] | 4501 | 0.113 | 0.2232 | No |
| 209 | NUDT2 | nudix hydrolase 2 [Source:HGNC Symbol;Acc:HGNC:8049] | 4521 | 0.112 | 0.2224 | No |
| 210 | STT3A | STT3 oligosaccharyltransferase complex catalytic subunit A [Source:HGNC Symbol;Acc:HGNC:6172] | 4618 | 0.103 | 0.2154 | No |
| 211 | NFKB1 | nuclear factor kappa B subunit 1 [Source:HGNC Symbol;Acc:HGNC:7794] | 4626 | 0.102 | 0.2156 | No |
| 212 | CHST11 | carbohydrate sulfotransferase 11 [Source:HGNC Symbol;Acc:HGNC:17422] | 4664 | 0.099 | 0.2133 | No |
| 213 | POMK | protein O-mannose kinase [Source:HGNC Symbol;Acc:HGNC:26267] | 4680 | 0.098 | 0.2127 | No |
| 214 | HS3ST3B1 | heparan sulfate-glucosamine 3-sulfotransferase 3B1 [Source:HGNC Symbol;Acc:HGNC:5198] | 4683 | 0.098 | 0.2132 | No |
| 215 | B3GAT1 | "beta-1,3-glucuronyltransferase 1 [Source:HGNC Symbol;Acc:HGNC:921]" | 4715 | 0.095 | 0.2114 | No |
| 216 | ATP7A | ATPase copper transporting alpha [Source:HGNC Symbol;Acc:HGNC:869] | 4814 | 0.086 | 0.2041 | No |
| 217 | PIGQ | phosphatidylinositol glycan anchor biosynthesis class Q [Source:HGNC Symbol;Acc:HGNC:14135] | 4825 | 0.085 | 0.2039 | No |
| 218 | MLYCD | malonyl-CoA decarboxylase [Source:HGNC Symbol;Acc:HGNC:7150] | 4852 | 0.083 | 0.2023 | No |
| 219 | DPM2 | "dolichyl-phosphate mannosyltransferase subunit 2, regulatory [Source:HGNC Symbol;Acc:HGNC:3006]" | 4862 | 0.083 | 0.2022 | No |
| 220 | POFUT1 | protein O-fucosyltransferase 1 [Source:HGNC Symbol;Acc:HGNC:14988] | 4910 | 0.079 | 0.1989 | No |
| 221 | TCF7L2 | transcription factor 7 like 2 [Source:HGNC Symbol;Acc:HGNC:11641] | 5034 | 0.070 | 0.1895 | No |
| 222 | RFK | riboflavin kinase [Source:HGNC Symbol;Acc:HGNC:30324] | 5071 | 0.067 | 0.1871 | No |
| 223 | MGAT5 | "alpha-1,6-mannosylglycoprotein 6-beta-N-acetylglucosaminyltransferase [Source:HGNC Symbol;Acc:HGNC:7049]" | 5086 | 0.066 | 0.1864 | No |
| 224 | B3GALT5 | "beta-1,3-galactosyltransferase 5 [Source:HGNC Symbol;Acc:HGNC:920]" | 5132 | 0.061 | 0.1832 | No |
| 225 | DPY19L4 | dpy-19 like 4 [Source:HGNC Symbol;Acc:HGNC:27829] | 5133 | 0.061 | 0.1836 | No |
| 226 | CHST3 | carbohydrate sulfotransferase 3 [Source:HGNC Symbol;Acc:HGNC:1971] | 5169 | 0.058 | 0.1812 | No |
| 227 | ADCY9 | adenylate cyclase 9 [Source:HGNC Symbol;Acc:HGNC:240] | 5191 | 0.057 | 0.1799 | No |
| 228 | TRIP11 | thyroid hormone receptor interactor 11 [Source:HGNC Symbol;Acc:HGNC:12305] | 5233 | 0.054 | 0.1770 | No |
| 229 | CHST10 | carbohydrate sulfotransferase 10 [Source:HGNC Symbol;Acc:HGNC:19650] | 5244 | 0.052 | 0.1765 | No |
| 230 | DHODH | dihydroorotate dehydrogenase (quinone) [Source:HGNC Symbol;Acc:HGNC:2867] | 5284 | 0.049 | 0.1737 | No |
| 231 | PGM3 | phosphoglucomutase 3 [Source:HGNC Symbol;Acc:HGNC:8907] | 5290 | 0.049 | 0.1737 | No |
| 232 | MGAT2 | "alpha-1,6-mannosyl-glycoprotein 2-beta-N-acetylglucosaminyltransferase [Source:HGNC Symbol;Acc:HGNC:7045]" | 5317 | 0.046 | 0.1719 | No |
| 233 | HACD1 | 3-hydroxyacyl-CoA dehydratase 1 [Source:HGNC Symbol;Acc:HGNC:9639] | 5342 | 0.044 | 0.1703 | No |
| 234 | ABCA2 | ATP binding cassette subfamily A member 2 [Source:HGNC Symbol;Acc:HGNC:32] | 5349 | 0.044 | 0.1701 | No |
| 235 | DOLPP1 | dolichyldiphosphatase 1 [Source:HGNC Symbol;Acc:HGNC:29565] | 5369 | 0.043 | 0.1688 | No |
| 236 | PANK4 | pantothenate kinase 4 (inactive) [Source:HGNC Symbol;Acc:HGNC:19366] | 5382 | 0.041 | 0.1681 | No |
| 237 | MGAT1 | "alpha-1,3-mannosyl-glycoprotein 2-beta-N-acetylglucosaminyltransferase [Source:HGNC Symbol;Acc:HGNC:7044]" | 5407 | 0.040 | 0.1665 | No |
| 238 | CMPK1 | cytidine/uridine monophosphate kinase 1 [Source:HGNC Symbol;Acc:HGNC:18170] | 5408 | 0.039 | 0.1668 | No |
| 239 | NDST3 | N-deacetylase and N-sulfotransferase 3 [Source:HGNC Symbol;Acc:HGNC:7682] | 5446 | 0.037 | 0.1640 | No |
| 240 | FA2H | fatty acid 2-hydroxylase [Source:HGNC Symbol;Acc:HGNC:21197] | 5466 | 0.035 | 0.1627 | No |
| 241 | NPC1 | NPC intracellular cholesterol transporter 1 [Source:HGNC Symbol;Acc:HGNC:7897] | 5480 | 0.034 | 0.1619 | No |
| 242 | SLC25A12 | solute carrier family 25 member 12 [Source:HGNC Symbol;Acc:HGNC:10982] | 5506 | 0.032 | 0.1601 | No |
| 243 | BCL2 | BCL2 apoptosis regulator [Source:HGNC Symbol;Acc:HGNC:990] | 5609 | 0.023 | 0.1521 | No |
| 244 | GALNT4 | polypeptide N-acetylgalactosaminyltransferase 4 [Source:HGNC Symbol;Acc:HGNC:4126] | 5610 | 0.023 | 0.1523 | No |
| 245 | DLAT | dihydrolipoamide S-acetyltransferase [Source:HGNC Symbol;Acc:HGNC:2896] | 5647 | 0.020 | 0.1495 | No |
| 246 | CCR7 | C-C motif chemokine receptor 7 [Source:HGNC Symbol;Acc:HGNC:1608] | 5678 | 0.017 | 0.1472 | No |
| 247 | ADSL | adenylosuccinate lyase [Source:HGNC Symbol;Acc:HGNC:291] | 5685 | 0.017 | 0.1468 | No |
| 248 | GFPT1 | glutamine--fructose-6-phosphate transaminase 1 [Source:HGNC Symbol;Acc:HGNC:4241] | 5847 | 0.005 | 0.1339 | No |
| 249 | GFPT2 | glutamine-fructose-6-phosphate transaminase 2 [Source:HGNC Symbol;Acc:HGNC:4242] | 5849 | 0.005 | 0.1339 | No |
| 250 | B3GALT1 | "beta-1,3-galactosyltransferase 1 [Source:HGNC Symbol;Acc:HGNC:916]" | 5864 | 0.004 | 0.1328 | No |
| 251 | UGT8 | UDP glycosyltransferase 8 [Source:HGNC Symbol;Acc:HGNC:12555] | 5908 | 0.002 | 0.1293 | No |
| 252 | GALNT6 | polypeptide N-acetylgalactosaminyltransferase 6 [Source:HGNC Symbol;Acc:HGNC:4128] | 5915 | 0.001 | 0.1289 | No |
| 253 | MGAT4B | "alpha-1,3-mannosyl-glycoprotein 4-beta-N-acetylglucosaminyltransferase B [Source:HGNC Symbol;Acc:HGNC:7048]" | 5956 | -0.002 | 0.1257 | No |
| 254 | BMPR1B | bone morphogenetic protein receptor type 1B [Source:HGNC Symbol;Acc:HGNC:1077] | 5959 | -0.002 | 0.1255 | No |
| 255 | ACSBG2 | acyl-CoA synthetase bubblegum family member 2 [Source:HGNC Symbol;Acc:HGNC:24174] | 5970 | -0.002 | 0.1247 | No |
| 256 | DUT | deoxyuridine triphosphatase [Source:HGNC Symbol;Acc:HGNC:3078] | 5981 | -0.003 | 0.1239 | No |
| 257 | RXYLT1 | ribitol xylosyltransferase 1 [Source:HGNC Symbol;Acc:HGNC:13530] | 6030 | -0.007 | 0.1201 | No |
| 258 | EDEM3 | ER degradation enhancing alpha-mannosidase like protein 3 [Source:HGNC Symbol;Acc:HGNC:16787] | 6073 | -0.010 | 0.1168 | No |
| 259 | ATP5PF | ATP synthase peripheral stalk subunit F6 [Source:HGNC Symbol;Acc:HGNC:847] | 6109 | -0.013 | 0.1141 | No |
| 260 | GLCE | glucuronic acid epimerase [Source:HGNC Symbol;Acc:HGNC:17855] | 6148 | -0.016 | 0.1111 | No |
| 261 | HACD2 | 3-hydroxyacyl-CoA dehydratase 2 [Source:HGNC Symbol;Acc:HGNC:9640] | 6162 | -0.017 | 0.1102 | No |
| 262 | EXTL1 | exostosin like glycosyltransferase 1 [Source:HGNC Symbol;Acc:HGNC:3515] | 6193 | -0.020 | 0.1079 | No |
| 263 | GALNT1 | polypeptide N-acetylgalactosaminyltransferase 1 [Source:HGNC Symbol;Acc:HGNC:4123] | 6217 | -0.022 | 0.1062 | No |
| 264 | NME1 | NME/NM23 nucleoside diphosphate kinase 1 [Source:HGNC Symbol;Acc:HGNC:7849] | 6328 | -0.031 | 0.0976 | No |
| 265 | CAD | "carbamoyl-phosphate synthetase 2, aspartate transcarbamylase, and dihydroorotase [Source:HGNC Symbol;Acc:HGNC:1424]" | 6359 | -0.032 | 0.0954 | No |
| 266 | ATP5ME | ATP synthase membrane subunit e [Source:HGNC Symbol;Acc:HGNC:846] | 6365 | -0.032 | 0.0952 | No |
| 267 | ATP5F1E | ATP synthase F1 subunit epsilon [Source:HGNC Symbol;Acc:HGNC:838] | 6405 | -0.036 | 0.0923 | No |
| 268 | HBEGF | heparin binding EGF like growth factor [Source:HGNC Symbol;Acc:HGNC:3059] | 6409 | -0.036 | 0.0923 | No |
| 269 | NUS1 | NUS1 dehydrodolichyl diphosphate synthase subunit [Source:HGNC Symbol;Acc:HGNC:21042] | 6415 | -0.037 | 0.0922 | No |
| 270 | SLC39A8 | solute carrier family 39 member 8 [Source:HGNC Symbol;Acc:HGNC:20862] | 6450 | -0.039 | 0.0897 | No |
| 271 | PIGG | phosphatidylinositol glycan anchor biosynthesis class G [Source:HGNC Symbol;Acc:HGNC:25985] | 6452 | -0.039 | 0.0899 | No |
| 272 | CHST7 | carbohydrate sulfotransferase 7 [Source:HGNC Symbol;Acc:HGNC:13817] | 6486 | -0.041 | 0.0875 | No |
| 273 | ACSL6 | acyl-CoA synthetase long chain family member 6 [Source:HGNC Symbol;Acc:HGNC:16496] | 6505 | -0.043 | 0.0864 | No |
| 274 | SLC25A1 | solute carrier family 25 member 1 [Source:HGNC Symbol;Acc:HGNC:10979] | 6509 | -0.043 | 0.0864 | No |
| 275 | GUCY2F | "guanylate cyclase 2F, retinal [Source:HGNC Symbol;Acc:HGNC:4691]" | 6510 | -0.043 | 0.0867 | No |
| 276 | GORASP1 | golgi reassembly stacking protein 1 [Source:HGNC Symbol;Acc:HGNC:16769] | 6526 | -0.044 | 0.0858 | No |
| 277 | SMPD3 | sphingomyelin phosphodiesterase 3 [Source:HGNC Symbol;Acc:HGNC:14240] | 6533 | -0.045 | 0.0856 | No |
| 278 | ST8SIA5 | "ST8 alpha-N-acetyl-neuraminide alpha-2,8-sialyltransferase 5 [Source:HGNC Symbol;Acc:HGNC:17827]" | 6565 | -0.047 | 0.0835 | No |
| 279 | RPN1 | ribophorin I [Source:HGNC Symbol;Acc:HGNC:10381] | 6618 | -0.051 | 0.0796 | No |
| 280 | ADCY8 | adenylate cyclase 8 [Source:HGNC Symbol;Acc:HGNC:239] | 6646 | -0.053 | 0.0778 | No |
| 281 | CWH43 | cell wall biogenesis 43 C-terminal homolog [Source:HGNC Symbol;Acc:HGNC:26133] | 6648 | -0.053 | 0.0781 | No |
| 282 | POMT1 | protein O-mannosyltransferase 1 [Source:HGNC Symbol;Acc:HGNC:9202] | 6652 | -0.053 | 0.0782 | No |
| 283 | IL4 | interleukin 4 [Source:HGNC Symbol;Acc:HGNC:6014] | 6673 | -0.054 | 0.0770 | No |
| 284 | GUCY1A2 | guanylate cyclase 1 soluble subunit alpha 2 [Source:HGNC Symbol;Acc:HGNC:4684] | 6731 | -0.058 | 0.0728 | No |
| 285 | PDHX | pyruvate dehydrogenase complex component X [Source:HGNC Symbol;Acc:HGNC:21350] | 6760 | -0.060 | 0.0709 | No |
| 286 | GALNT14 | polypeptide N-acetylgalactosaminyltransferase 14 [Source:HGNC Symbol;Acc:HGNC:22946] | 6762 | -0.060 | 0.0713 | No |
| 287 | NPR2 | natriuretic peptide receptor 2 [Source:HGNC Symbol;Acc:HGNC:7944] | 6771 | -0.061 | 0.0710 | No |
| 288 | CHST2 | carbohydrate sulfotransferase 2 [Source:HGNC Symbol;Acc:HGNC:1970] | 6778 | -0.061 | 0.0710 | No |
| 289 | CCL21 | C-C motif chemokine ligand 21 [Source:HGNC Symbol;Acc:HGNC:10620] | 6856 | -0.068 | 0.0652 | No |
| 290 | PDGFRB | platelet derived growth factor receptor beta [Source:HGNC Symbol;Acc:HGNC:8804] | 6914 | -0.073 | 0.0612 | No |
| 291 | HPRT1 | hypoxanthine phosphoribosyltransferase 1 [Source:HGNC Symbol;Acc:HGNC:5157] | 6954 | -0.076 | 0.0585 | No |
| 292 | XYLB | xylulokinase [Source:HGNC Symbol;Acc:HGNC:12839] | 7054 | -0.084 | 0.0512 | No |
| 293 | FKRP | fukutin related protein [Source:HGNC Symbol;Acc:HGNC:17997] | 7065 | -0.085 | 0.0509 | No |
| 294 | G6PD | glucose-6-phosphate dehydrogenase [Source:HGNC Symbol;Acc:HGNC:4057] | 7082 | -0.086 | 0.0502 | No |
| 295 | OGA | O-GlcNAcase [Source:HGNC Symbol;Acc:HGNC:7056] | 7086 | -0.086 | 0.0506 | No |
| 296 | ADCY10 | adenylate cyclase 10 [Source:HGNC Symbol;Acc:HGNC:21285] | 7096 | -0.087 | 0.0504 | No |
| 297 | LARGE1 | LARGE xylosyl- and glucuronyltransferase 1 [Source:HGNC Symbol;Acc:HGNC:6511] | 7164 | -0.093 | 0.0457 | No |
| 298 | NECAB3 | N-terminal EF-hand calcium binding protein 3 [Source:HGNC Symbol;Acc:HGNC:15851] | 7224 | -0.097 | 0.0416 | No |
| 299 | GFUS | GDP-L-fucose synthase [Source:HGNC Symbol;Acc:HGNC:12390] | 7317 | -0.103 | 0.0349 | No |
| 300 | SRD5A3 | steroid 5 alpha-reductase 3 [Source:HGNC Symbol;Acc:HGNC:25812] | 7325 | -0.104 | 0.0351 | No |
| 301 | DPAGT1 | dolichyl-phosphate N-acetylglucosaminephosphotransferase 1 [Source:HGNC Symbol;Acc:HGNC:2995] | 7452 | -0.115 | 0.0257 | No |
| 302 | GALNT2 | polypeptide N-acetylgalactosaminyltransferase 2 [Source:HGNC Symbol;Acc:HGNC:4124] | 7453 | -0.115 | 0.0265 | No |
| 303 | TK2 | thymidine kinase 2 [Source:HGNC Symbol;Acc:HGNC:11831] | 7468 | -0.116 | 0.0262 | No |
| 304 | DSE | dermatan sulfate epimerase [Source:HGNC Symbol;Acc:HGNC:21144] | 7493 | -0.118 | 0.0250 | No |
| 305 | NANS | N-acetylneuraminate synthase [Source:HGNC Symbol;Acc:HGNC:19237] | 7530 | -0.121 | 0.0230 | No |
| 306 | SOAT1 | sterol O-acyltransferase 1 [Source:HGNC Symbol;Acc:HGNC:11177] | 7555 | -0.124 | 0.0219 | No |
| 307 | LDHC | lactate dehydrogenase C [Source:HGNC Symbol;Acc:HGNC:6544] | 7603 | -0.127 | 0.0190 | No |
| 308 | SLC26A1 | solute carrier family 26 member 1 [Source:HGNC Symbol;Acc:HGNC:10993] | 7618 | -0.128 | 0.0187 | No |
| 309 | PDK3 | pyruvate dehydrogenase kinase 3 [Source:HGNC Symbol;Acc:HGNC:8811] | 7661 | -0.132 | 0.0162 | No |
| 310 | DPM1 | "dolichyl-phosphate mannosyltransferase subunit 1, catalytic [Source:HGNC Symbol;Acc:HGNC:3005]" | 7723 | -0.135 | 0.0122 | No |
| 311 | B4GALNT1 | "beta-1,4-N-acetyl-galactosaminyltransferase 1 [Source:HGNC Symbol;Acc:HGNC:4117]" | 7799 | -0.141 | 0.0072 | No |
| 312 | UAP1L1 | UDP-N-acetylglucosamine pyrophosphorylase 1 like 1 [Source:HGNC Symbol;Acc:HGNC:28082] | 7909 | -0.148 | -0.0006 | No |
| 313 | FOXL1 | forkhead box L1 [Source:HGNC Symbol;Acc:HGNC:3817] | 7919 | -0.149 | -0.0003 | No |
| 314 | CHPF | chondroitin polymerizing factor [Source:HGNC Symbol;Acc:HGNC:24291] | 7960 | -0.152 | -0.0025 | No |
| 315 | ATP6V1A | ATPase H+ transporting V1 subunit A [Source:HGNC Symbol;Acc:HGNC:851] | 8013 | -0.156 | -0.0056 | No |
| 316 | ELOVL2 | ELOVL fatty acid elongase 2 [Source:HGNC Symbol;Acc:HGNC:14416] | 8080 | -0.161 | -0.0098 | No |
| 317 | ADA | adenosine deaminase [Source:HGNC Symbol;Acc:HGNC:186] | 8105 | -0.163 | -0.0106 | No |
| 318 | GALNT8 | polypeptide N-acetylgalactosaminyltransferase 8 [Source:HGNC Symbol;Acc:HGNC:4130] | 8124 | -0.164 | -0.0109 | No |
| 319 | HS3ST1 | heparan sulfate-glucosamine 3-sulfotransferase 1 [Source:HGNC Symbol;Acc:HGNC:5194] | 8162 | -0.168 | -0.0128 | No |
| 320 | ACSL3 | acyl-CoA synthetase long chain family member 3 [Source:HGNC Symbol;Acc:HGNC:3570] | 8170 | -0.169 | -0.0122 | No |
| 321 | ACLY | ATP citrate lyase [Source:HGNC Symbol;Acc:HGNC:115] | 8173 | -0.169 | -0.0112 | No |
| 322 | GCDH | glutaryl-CoA dehydrogenase [Source:HGNC Symbol;Acc:HGNC:4189] | 8180 | -0.170 | -0.0105 | No |
| 323 | CHST15 | carbohydrate sulfotransferase 15 [Source:HGNC Symbol;Acc:HGNC:18137] | 8220 | -0.173 | -0.0125 | No |
| 324 | CTPS2 | CTP synthase 2 [Source:HGNC Symbol;Acc:HGNC:2520] | 8238 | -0.174 | -0.0127 | No |
| 325 | CDA | cytidine deaminase [Source:HGNC Symbol;Acc:HGNC:1712] | 8324 | -0.180 | -0.0183 | No |
| 326 | MPDU1 | mannose-P-dolichol utilization defect 1 [Source:HGNC Symbol;Acc:HGNC:7207] | 8330 | -0.180 | -0.0175 | No |
| 327 | B3GNT3 | "UDP-GlcNAc:betaGal beta-1,3-N-acetylglucosaminyltransferase 3 [Source:HGNC Symbol;Acc:HGNC:13528]" | 8361 | -0.183 | -0.0186 | No |
| 328 | GAL3ST1 | galactose-3-O-sulfotransferase 1 [Source:HGNC Symbol;Acc:HGNC:24240] | 8378 | -0.184 | -0.0187 | No |
| 329 | PRPS1L1 | phosphoribosyl pyrophosphate synthetase 1 like 1 [Source:HGNC Symbol;Acc:HGNC:9463] | 8382 | -0.184 | -0.0177 | No |
| 330 | ELOVL1 | ELOVL fatty acid elongase 1 [Source:HGNC Symbol;Acc:HGNC:14418] | 8406 | -0.186 | -0.0182 | No |
| 331 | CHST8 | carbohydrate sulfotransferase 8 [Source:HGNC Symbol;Acc:HGNC:15993] | 8526 | -0.196 | -0.0265 | No |
| 332 | UGGT2 | UDP-glucose glycoprotein glucosyltransferase 2 [Source:HGNC Symbol;Acc:HGNC:15664] | 8568 | -0.199 | -0.0284 | No |
| 333 | ADCY3 | adenylate cyclase 3 [Source:HGNC Symbol;Acc:HGNC:234] | 8572 | -0.199 | -0.0273 | No |
| 334 | GCNT2 | glucosaminyl (N-acetyl) transferase 2 (I blood group) [Source:HGNC Symbol;Acc:HGNC:4204] | 8619 | -0.202 | -0.0296 | No |
| 335 | VEGFB | vascular endothelial growth factor B [Source:HGNC Symbol;Acc:HGNC:12681] | 8673 | -0.206 | -0.0325 | No |
| 336 | CYTL1 | cytokine like 1 [Source:HGNC Symbol;Acc:HGNC:24435] | 8685 | -0.208 | -0.0319 | No |
| 337 | SLC25A13 | solute carrier family 25 member 13 [Source:HGNC Symbol;Acc:HGNC:10983] | 8713 | -0.210 | -0.0327 | No |
| 338 | SLC35D2 | solute carrier family 35 member D2 [Source:HGNC Symbol;Acc:HGNC:20799] | 8714 | -0.210 | -0.0313 | No |
| 339 | PAWR | pro-apoptotic WT1 regulator [Source:HGNC Symbol;Acc:HGNC:8614] | 8766 | -0.213 | -0.0339 | No |
| 340 | FLCN | folliculin [Source:HGNC Symbol;Acc:HGNC:27310] | 8848 | -0.221 | -0.0389 | No |
| 341 | ABCA7 | ATP binding cassette subfamily A member 7 [Source:HGNC Symbol;Acc:HGNC:37] | 8856 | -0.222 | -0.0380 | No |
| 342 | ST6GALNAC2 | "ST6 N-acetylgalactosaminide alpha-2,6-sialyltransferase 2 [Source:HGNC Symbol;Acc:HGNC:10867]" | 8870 | -0.222 | -0.0375 | No |
| 343 | FUT5 | fucosyltransferase 5 [Source:HGNC Symbol;Acc:HGNC:4016] | 8898 | -0.225 | -0.0381 | No |
| 344 | MGAT3 | "beta-1,4-mannosyl-glycoprotein 4-beta-N-acetylglucosaminyltransferase [Source:HGNC Symbol;Acc:HGNC:7046]" | 8920 | -0.226 | -0.0383 | No |
| 345 | AATF | apoptosis antagonizing transcription factor [Source:HGNC Symbol;Acc:HGNC:19235] | 8935 | -0.227 | -0.0379 | No |
| 346 | PIGZ | phosphatidylinositol glycan anchor biosynthesis class Z [Source:HGNC Symbol;Acc:HGNC:30596] | 9047 | -0.235 | -0.0452 | No |
| 347 | B4GALT5 | "beta-1,4-galactosyltransferase 5 [Source:HGNC Symbol;Acc:HGNC:928]" | 9051 | -0.236 | -0.0438 | No |
| 348 | ABCC5 | ATP binding cassette subfamily C member 5 [Source:HGNC Symbol;Acc:HGNC:56] | 9066 | -0.237 | -0.0433 | No |
| 349 | IL33 | interleukin 33 [Source:HGNC Symbol;Acc:HGNC:16028] | 9078 | -0.238 | -0.0426 | No |
| 350 | PDHA2 | pyruvate dehydrogenase E1 subunit alpha 2 [Source:HGNC Symbol;Acc:HGNC:8807] | 9124 | -0.241 | -0.0446 | No |
| 351 | ACSL5 | acyl-CoA synthetase long chain family member 5 [Source:HGNC Symbol;Acc:HGNC:16526] | 9136 | -0.243 | -0.0438 | No |
| 352 | ACACA | acetyl-CoA carboxylase alpha [Source:HGNC Symbol;Acc:HGNC:84] | 9177 | -0.246 | -0.0454 | No |
| 353 | POMT2 | protein O-mannosyltransferase 2 [Source:HGNC Symbol;Acc:HGNC:19743] | 9216 | -0.249 | -0.0467 | No |
| 354 | PANK3 | pantothenate kinase 3 [Source:HGNC Symbol;Acc:HGNC:19365] | 9286 | -0.255 | -0.0505 | No |
| 355 | SERP1 | stress associated endoplasmic reticulum protein 1 [Source:HGNC Symbol;Acc:HGNC:10759] | 9343 | -0.259 | -0.0533 | No |
| 356 | PGAP4 | post-GPI attachment to proteins GalNAc transferase 4 [Source:HGNC Symbol;Acc:HGNC:28180] | 9347 | -0.259 | -0.0517 | No |
| 357 | PGAP1 | post-GPI attachment to proteins inositol deacylase 1 [Source:HGNC Symbol;Acc:HGNC:25712] | 9358 | -0.260 | -0.0508 | No |
| 358 | PMM2 | phosphomannomutase 2 [Source:HGNC Symbol;Acc:HGNC:9115] | 9408 | -0.265 | -0.0529 | No |
| 359 | ENTPD5 | ectonucleoside triphosphate diphosphohydrolase 5 (inactive) [Source:HGNC Symbol;Acc:HGNC:3367] | 9414 | -0.265 | -0.0515 | No |
| 360 | GUCY1A1 | guanylate cyclase 1 soluble subunit alpha 1 [Source:HGNC Symbol;Acc:HGNC:4685] | 9420 | -0.266 | -0.0501 | No |
| 361 | FUT9 | fucosyltransferase 9 [Source:HGNC Symbol;Acc:HGNC:4020] | 9433 | -0.268 | -0.0492 | No |
| 362 | HS3ST2 | heparan sulfate-glucosamine 3-sulfotransferase 2 [Source:HGNC Symbol;Acc:HGNC:5195] | 9469 | -0.271 | -0.0502 | No |
| 363 | PAICS | phosphoribosylaminoimidazole carboxylase and phosphoribosylaminoimidazolesuccinocarboxamide synthase [Source:HGNC Symbol;Acc:HGNC:8587] | 9475 | -0.272 | -0.0488 | No |
| 364 | B3GNT4 | "UDP-GlcNAc:betaGal beta-1,3-N-acetylglucosaminyltransferase 4 [Source:HGNC Symbol;Acc:HGNC:15683]" | 9500 | -0.274 | -0.0488 | No |
| 365 | UGGT1 | UDP-glucose glycoprotein glucosyltransferase 1 [Source:HGNC Symbol;Acc:HGNC:15663] | 9516 | -0.275 | -0.0482 | No |
| 366 | SLC26A2 | solute carrier family 26 member 2 [Source:HGNC Symbol;Acc:HGNC:10994] | 9523 | -0.276 | -0.0468 | No |
| 367 | AMPD2 | adenosine monophosphate deaminase 2 [Source:HGNC Symbol;Acc:HGNC:469] | 9530 | -0.276 | -0.0454 | No |
| 368 | PIGA | phosphatidylinositol glycan anchor biosynthesis class A [Source:HGNC Symbol;Acc:HGNC:8957] | 9571 | -0.280 | -0.0467 | No |
| 369 | ENO1 | enolase 1 [Source:HGNC Symbol;Acc:HGNC:3350] | 9606 | -0.284 | -0.0475 | No |
| 370 | NPR1 | natriuretic peptide receptor 1 [Source:HGNC Symbol;Acc:HGNC:7943] | 9634 | -0.286 | -0.0477 | No |
| 371 | PNP | purine nucleoside phosphorylase [Source:HGNC Symbol;Acc:HGNC:7892] | 9657 | -0.288 | -0.0475 | No |
| 372 | AK4 | adenylate kinase 4 [Source:HGNC Symbol;Acc:HGNC:363] | 9682 | -0.290 | -0.0475 | No |
| 373 | ADCY2 | adenylate cyclase 2 [Source:HGNC Symbol;Acc:HGNC:233] | 9715 | -0.293 | -0.0480 | No |
| 374 | SHMT1 | serine hydroxymethyltransferase 1 [Source:HGNC Symbol;Acc:HGNC:10850] | 9727 | -0.294 | -0.0469 | No |
| 375 | CHST5 | carbohydrate sulfotransferase 5 [Source:HGNC Symbol;Acc:HGNC:1973] | 9732 | -0.295 | -0.0452 | No |
| 376 | FKTN | fukutin [Source:HGNC Symbol;Acc:HGNC:3622] | 9745 | -0.296 | -0.0442 | No |
| 377 | A4GALT | "alpha 1,4-galactosyltransferase (P blood group) [Source:HGNC Symbol;Acc:HGNC:18149]" | 9766 | -0.297 | -0.0438 | No |
| 378 | CSGALNACT2 | chondroitin sulfate N-acetylgalactosaminyltransferase 2 [Source:HGNC Symbol;Acc:HGNC:24292] | 9773 | -0.298 | -0.0422 | No |
| 379 | POGLUT1 | protein O-glucosyltransferase 1 [Source:HGNC Symbol;Acc:HGNC:22954] | 9794 | -0.301 | -0.0418 | No |
| 380 | BACE2 | beta-secretase 2 [Source:HGNC Symbol;Acc:HGNC:934] | 9838 | -0.305 | -0.0432 | No |
| 381 | GART | "phosphoribosylglycinamide formyltransferase, phosphoribosylglycinamide synthetase, phosphoribosylaminoimidazole synthetase [Source:HGNC Symbol;Acc:HGNC:4163]" | 9861 | -0.307 | -0.0428 | No |
| 382 | FUT3 | fucosyltransferase 3 (Lewis blood group) [Source:HGNC Symbol;Acc:HGNC:4014] | 9890 | -0.311 | -0.0430 | No |
| 383 | CHST4 | carbohydrate sulfotransferase 4 [Source:HGNC Symbol;Acc:HGNC:1972] | 9941 | -0.315 | -0.0448 | No |
| 384 | TET3 | tet methylcytosine dioxygenase 3 [Source:HGNC Symbol;Acc:HGNC:28313] | 9962 | -0.317 | -0.0443 | No |
| 385 | ST8SIA3 | "ST8 alpha-N-acetyl-neuraminide alpha-2,8-sialyltransferase 3 [Source:HGNC Symbol;Acc:HGNC:14269]" | 10046 | -0.326 | -0.0487 | No |
| 386 | PLOD2 | "procollagen-lysine,2-oxoglutarate 5-dioxygenase 2 [Source:HGNC Symbol;Acc:HGNC:9082]" | 10086 | -0.330 | -0.0496 | No |
| 387 | PPT2 | palmitoyl-protein thioesterase 2 [Source:HGNC Symbol;Acc:HGNC:9326] | 10103 | -0.331 | -0.0487 | No |
| 388 | PKLR | pyruvate kinase L/R [Source:HGNC Symbol;Acc:HGNC:9020] | 10120 | -0.333 | -0.0477 | No |
| 389 | EOGT | EGF domain specific O-linked N-acetylglucosamine transferase [Source:HGNC Symbol;Acc:HGNC:28526] | 10174 | -0.338 | -0.0496 | No |
| 390 | MAN1A1 | mannosidase alpha class 1A member 1 [Source:HGNC Symbol;Acc:HGNC:6821] | 10207 | -0.341 | -0.0499 | No |
| 391 | FAM20B | FAM20B glycosaminoglycan xylosylkinase [Source:HGNC Symbol;Acc:HGNC:23017] | 10227 | -0.344 | -0.0491 | No |
| 392 | UGDH | UDP-glucose 6-dehydrogenase [Source:HGNC Symbol;Acc:HGNC:12525] | 10279 | -0.349 | -0.0508 | No |
| 393 | GOLGA2 | golgin A2 [Source:HGNC Symbol;Acc:HGNC:4425] | 10285 | -0.349 | -0.0488 | No |
| 394 | GK | glycerol kinase [Source:HGNC Symbol;Acc:HGNC:4289] | 10291 | -0.350 | -0.0468 | No |
| 395 | GALNT12 | polypeptide N-acetylgalactosaminyltransferase 12 [Source:HGNC Symbol;Acc:HGNC:19877] | 10414 | -0.362 | -0.0542 | No |
| 396 | AMD1 | adenosylmethionine decarboxylase 1 [Source:HGNC Symbol;Acc:HGNC:457] | 10415 | -0.363 | -0.0517 | No |
| 397 | TAFAZZIN | "tafazzin, phospholipid-lysophospholipid transacylase [Source:HGNC Symbol;Acc:HGNC:11577]" | 10429 | -0.364 | -0.0503 | No |
| 398 | ARFGEF1 | ADP ribosylation factor guanine nucleotide exchange factor 1 [Source:HGNC Symbol;Acc:HGNC:15772] | 10477 | -0.371 | -0.0515 | No |
| 399 | UCK2 | uridine-cytidine kinase 2 [Source:HGNC Symbol;Acc:HGNC:12562] | 10481 | -0.371 | -0.0492 | No |
| 400 | B4GALT2 | "beta-1,4-galactosyltransferase 2 [Source:HGNC Symbol;Acc:HGNC:925]" | 10490 | -0.372 | -0.0474 | No |
| 401 | PPARGC1A | PPARG coactivator 1 alpha [Source:HGNC Symbol;Acc:HGNC:9237] | 10504 | -0.374 | -0.0459 | No |
| 402 | CTPS1 | CTP synthase 1 [Source:HGNC Symbol;Acc:HGNC:2519] | 10569 | -0.380 | -0.0484 | No |
| 403 | MOGS | mannosyl-oligosaccharide glucosidase [Source:HGNC Symbol;Acc:HGNC:24862] | 10630 | -0.386 | -0.0506 | No |
| 404 | ST3GAL4 | "ST3 beta-galactoside alpha-2,3-sialyltransferase 4 [Source:HGNC Symbol;Acc:HGNC:10864]" | 10688 | -0.391 | -0.0525 | No |
| 405 | ACSL1 | acyl-CoA synthetase long chain family member 1 [Source:HGNC Symbol;Acc:HGNC:3569] | 10705 | -0.394 | -0.0511 | No |
| 406 | TMSB4X | thymosin beta 4 X-linked [Source:HGNC Symbol;Acc:HGNC:11881] | 10741 | -0.398 | -0.0512 | No |
| 407 | SLC2A4 | solute carrier family 2 member 4 [Source:HGNC Symbol;Acc:HGNC:11009] | 10764 | -0.400 | -0.0503 | No |
| 408 | JAK3 | Janus kinase 3 [Source:HGNC Symbol;Acc:HGNC:6193] | 10908 | -0.418 | -0.0589 | No |
| 409 | CHP1 | calcineurin like EF-hand protein 1 [Source:HGNC Symbol;Acc:HGNC:17433] | 10940 | -0.423 | -0.0585 | No |
| 410 | PFAS | phosphoribosylformylglycinamidine synthase [Source:HGNC Symbol;Acc:HGNC:8863] | 10943 | -0.424 | -0.0558 | No |
| 411 | ACACB | acetyl-CoA carboxylase beta [Source:HGNC Symbol;Acc:HGNC:85] | 10955 | -0.425 | -0.0538 | No |
| 412 | GATA1 | GATA binding protein 1 [Source:HGNC Symbol;Acc:HGNC:4170] | 10958 | -0.425 | -0.0511 | No |
| 413 | HAS2 | hyaluronan synthase 2 [Source:HGNC Symbol;Acc:HGNC:4819] | 10980 | -0.428 | -0.0499 | No |
| 414 | IMPDH1 | inosine monophosphate dehydrogenase 1 [Source:HGNC Symbol;Acc:HGNC:6052] | 11023 | -0.433 | -0.0503 | No |
| 415 | GALNT3 | polypeptide N-acetylgalactosaminyltransferase 3 [Source:HGNC Symbol;Acc:HGNC:4125] | 11062 | -0.440 | -0.0503 | No |
| 416 | FUT1 | fucosyltransferase 1 (H blood group) [Source:HGNC Symbol;Acc:HGNC:4012] | 11086 | -0.442 | -0.0492 | No |
| 417 | PPARA | peroxisome proliferator activated receptor alpha [Source:HGNC Symbol;Acc:HGNC:9232] | 11110 | -0.445 | -0.0480 | No |
| 418 | UAP1 | UDP-N-acetylglucosamine pyrophosphorylase 1 [Source:HGNC Symbol;Acc:HGNC:12457] | 11131 | -0.447 | -0.0466 | No |
| 419 | ST6GALNAC4 | "ST6 N-acetylgalactosaminide alpha-2,6-sialyltransferase 4 [Source:HGNC Symbol;Acc:HGNC:17846]" | 11164 | -0.452 | -0.0461 | No |
| 420 | BMPR2 | bone morphogenetic protein receptor type 2 [Source:HGNC Symbol;Acc:HGNC:1078] | 11174 | -0.454 | -0.0437 | No |
| 421 | NDST1 | N-deacetylase and N-sulfotransferase 1 [Source:HGNC Symbol;Acc:HGNC:7680] | 11241 | -0.462 | -0.0458 | No |
| 422 | AGO2 | argonaute RISC catalytic component 2 [Source:HGNC Symbol;Acc:HGNC:3263] | 11246 | -0.463 | -0.0430 | No |
| 423 | ELOVL5 | ELOVL fatty acid elongase 5 [Source:HGNC Symbol;Acc:HGNC:21308] | 11272 | -0.467 | -0.0418 | No |
| 424 | SLC35C1 | solute carrier family 35 member C1 [Source:HGNC Symbol;Acc:HGNC:20197] | 11286 | -0.470 | -0.0397 | No |
| 425 | ACSL4 | acyl-CoA synthetase long chain family member 4 [Source:HGNC Symbol;Acc:HGNC:3571] | 11327 | -0.475 | -0.0397 | No |
| 426 | PDK4 | pyruvate dehydrogenase kinase 4 [Source:HGNC Symbol;Acc:HGNC:8812] | 11384 | -0.483 | -0.0409 | No |
| 427 | SLC25A16 | solute carrier family 25 member 16 [Source:HGNC Symbol;Acc:HGNC:10986] | 11411 | -0.488 | -0.0397 | No |
| 428 | FUT2 | fucosyltransferase 2 [Source:HGNC Symbol;Acc:HGNC:4013] | 11459 | -0.497 | -0.0401 | No |
| 429 | NPPB | natriuretic peptide B [Source:HGNC Symbol;Acc:HGNC:7940] | 11463 | -0.497 | -0.0369 | No |
| 430 | VPS9D1 | VPS9 domain containing 1 [Source:HGNC Symbol;Acc:HGNC:13526] | 11495 | -0.501 | -0.0360 | No |
| 431 | PRKAA1 | protein kinase AMP-activated catalytic subunit alpha 1 [Source:HGNC Symbol;Acc:HGNC:9376] | 11499 | -0.501 | -0.0328 | No |
| 432 | UCKL1 | uridine-cytidine kinase 1 like 1 [Source:HGNC Symbol;Acc:HGNC:15938] | 11515 | -0.503 | -0.0306 | No |
| 433 | B4GALT1 | "beta-1,4-galactosyltransferase 1 [Source:HGNC Symbol;Acc:HGNC:924]" | 11530 | -0.505 | -0.0283 | No |
| 434 | PPAT | phosphoribosyl pyrophosphate amidotransferase [Source:HGNC Symbol;Acc:HGNC:9238] | 11534 | -0.505 | -0.0251 | No |
| 435 | A4GNT | "alpha-1,4-N-acetylglucosaminyltransferase [Source:HGNC Symbol;Acc:HGNC:17968]" | 11568 | -0.510 | -0.0243 | No |
| 436 | TMEM165 | transmembrane protein 165 [Source:HGNC Symbol;Acc:HGNC:30760] | 11578 | -0.512 | -0.0215 | No |
| 437 | CHSY1 | chondroitin sulfate synthase 1 [Source:HGNC Symbol;Acc:HGNC:17198] | 11628 | -0.521 | -0.0219 | No |
| 438 | DCAKD | dephospho-CoA kinase domain containing [Source:HGNC Symbol;Acc:HGNC:26238] | 11650 | -0.525 | -0.0200 | No |
| 439 | NPPA | natriuretic peptide A [Source:HGNC Symbol;Acc:HGNC:7939] | 11653 | -0.525 | -0.0166 | No |
| 440 | ST3GAL2 | "ST3 beta-galactoside alpha-2,3-sialyltransferase 2 [Source:HGNC Symbol;Acc:HGNC:10863]" | 11721 | -0.536 | -0.0183 | No |
| 441 | POFUT2 | protein O-fucosyltransferase 2 [Source:HGNC Symbol;Acc:HGNC:14683] | 11725 | -0.537 | -0.0149 | No |
| 442 | B4GALT3 | "beta-1,4-galactosyltransferase 3 [Source:HGNC Symbol;Acc:HGNC:926]" | 11748 | -0.540 | -0.0130 | No |
| 443 | MAN2A2 | mannosidase alpha class 2A member 2 [Source:HGNC Symbol;Acc:HGNC:6825] | 11772 | -0.545 | -0.0111 | No |
| 444 | NECAB2 | N-terminal EF-hand calcium binding protein 2 [Source:HGNC Symbol;Acc:HGNC:23746] | 11795 | -0.548 | -0.0092 | No |
| 445 | PIGL | phosphatidylinositol glycan anchor biosynthesis class L [Source:HGNC Symbol;Acc:HGNC:8966] | 11817 | -0.553 | -0.0071 | No |
| 446 | HS6ST1 | heparan sulfate 6-O-sulfotransferase 1 [Source:HGNC Symbol;Acc:HGNC:5201] | 11932 | -0.579 | -0.0123 | No |
| 447 | ADCY6 | adenylate cyclase 6 [Source:HGNC Symbol;Acc:HGNC:237] | 12005 | -0.594 | -0.0141 | No |
| 448 | UPP1 | uridine phosphorylase 1 [Source:HGNC Symbol;Acc:HGNC:12576] | 12013 | -0.595 | -0.0106 | No |
| 449 | CHST1 | carbohydrate sulfotransferase 1 [Source:HGNC Symbol;Acc:HGNC:1969] | 12049 | -0.606 | -0.0093 | No |
| 450 | FUT6 | fucosyltransferase 6 [Source:HGNC Symbol;Acc:HGNC:4017] | 12067 | -0.612 | -0.0065 | No |
| 451 | ST3GAL1 | "ST3 beta-galactoside alpha-2,3-sialyltransferase 1 [Source:HGNC Symbol;Acc:HGNC:10862]" | 12092 | -0.619 | -0.0042 | No |
| 452 | IL1B | interleukin 1 beta [Source:HGNC Symbol;Acc:HGNC:5992] | 12105 | -0.622 | -0.0009 | No |
| 453 | ITM2B | integral membrane protein 2B [Source:HGNC Symbol;Acc:HGNC:6174] | 12252 | -0.662 | -0.0081 | No |
| 454 | TKT | transketolase [Source:HGNC Symbol;Acc:HGNC:11834] | 12373 | -0.700 | -0.0130 | No |
| 455 | ADSS2 | adenylosuccinate synthase 2 [Source:HGNC Symbol;Acc:HGNC:292] | 12376 | -0.701 | -0.0084 | No |
| 456 | PDGFB | platelet derived growth factor subunit B [Source:HGNC Symbol;Acc:HGNC:8800] | 12389 | -0.704 | -0.0046 | No |
| 457 | DIP2A | disco interacting protein 2 homolog A [Source:HGNC Symbol;Acc:HGNC:17217] | 12433 | -0.719 | -0.0031 | No |
| 458 | OGT | O-linked N-acetylglucosamine (GlcNAc) transferase [Source:HGNC Symbol;Acc:HGNC:8127] | 12440 | -0.721 | 0.0013 | No |
| 459 | EXTL3 | exostosin like glycosyltransferase 3 [Source:HGNC Symbol;Acc:HGNC:3518] | 12442 | -0.722 | 0.0061 | No |
| 460 | UGCG | UDP-glucose ceramide glucosyltransferase [Source:HGNC Symbol;Acc:HGNC:12524] | 12532 | -0.763 | 0.0042 | No |
| 461 | GNE | glucosamine (UDP-N-acetyl)-2-epimerase/N-acetylmannosamine kinase [Source:HGNC Symbol;Acc:HGNC:23657] | 12538 | -0.767 | 0.0090 | No |
| 462 | HAS1 | hyaluronan synthase 1 [Source:HGNC Symbol;Acc:HGNC:4818] | 12655 | -0.831 | 0.0053 | No |
| 463 | MYC | "MYC proto-oncogene, bHLH transcription factor [Source:HGNC Symbol;Acc:HGNC:7553]" | 12801 | -0.970 | 0.0003 | No |
| 464 | CTNNB1 | catenin beta 1 [Source:HGNC Symbol;Acc:HGNC:2514] | 12866 | -1.119 | 0.0027 | No |
Table: GSEA details [plain text format]

  

Fig 2: GOBP\_CARBOHYDRATE\_DERIVATIVE\_BIOSYNTHETIC\_PROCESS      
 Blue-Pink O' Gram in the Space of the Analyzed GeneSet

  

Fig 3: GOBP\_CARBOHYDRATE\_DERIVATIVE\_BIOSYNTHETIC\_PROCESS: Random ES distribution      
 Gene set null distribution of ES for **GOBP\_CARBOHYDRATE\_DERIVATIVE\_BIOSYNTHETIC\_PROCESS**
